# The Effects of Thai Herbal Ha-Rak Formula on COX Isoform Expression in Human Umbilical Vein Endothelial Cells Induced by IL-1*β*

**DOI:** 10.1155/2017/9383272

**Published:** 2017-10-29

**Authors:** Titchaporn Palo, Athiwat Thaworn, Phornnapa Charoenkij, Onusa Thamsermsang, Sirikul Chotewuttakorn, Pinpat Tripatara, Tawee Laohapand, Pravit Akarasereenont

**Affiliations:** ^1^Department of Pharmacology, Faculty of Medicine, Siriraj Hospital, Mahidol University, Bangkok 10700, Thailand; ^2^Center of Applied Thai Traditional Medicine, Faculty of Medicine, Siriraj Hospital, Mahidol University, Bangkok 10700, Thailand

## Abstract

**Objective:**

To investigate the modulated effects of HRF on cyclooxygenase isoform expression and its activity, using the human umbilical vein endothelial cell (HUVEC) model induced by interleukin-1 beta (IL-1*β*).

**Methods:**

Cells were treated with indomethacin (positive control), HRF, and its components at various concentrations prior to treatment with IL-1*β* at 24 h. Cell viability was determined by MTT assay. Moreover, the anti-inflammatory effects of HRF and its components through mRNA and protein expression were established using real-time quantitative PCR and Western blot, respectively. COX activity was identified via exogenous and endogenous PGE_2_ productions using the EIA.

**Result:**

There was no cytotoxicity in HUVECs treated with HRF. None of the experimental conditions used in the study affected the expression of COX-1, but COX-2 protein expression was inhibited at concentrations under 10 *µ*g/mL. Despite the significantly increased levels of exogenous PGE_2_, HRF had no effect on COX-2 mRNA expression. However, the production of PGE_2_ was lower at a concentration of 100 *µ*g/mL HRF than at a concentration below 10 *µ*g/mL. Interestingly, each component of HRF revealed different effects of the Ha-Rak formula.

**Conclusion:**

Our preliminary findings suggest that HRF and its components provide diverse modulation of COX-2 and PGE_2_ at the* in vitro* level.

## 1. Introduction

Fever, an excessively high body temperature, is a defensive mechanism of humans and is found as a clinical sign of inflammation [1]. Numerous endogenous and exogenous factors trigger a febrile response and lead to the release of various inflammatory mediators from immune and nonimmune cells, such as leukocytes, macrophages, and endothelial cells [2, 3]. These cells play an essential role in generating several proinflammatory cytokines, namely, interleukin-1 (IL-1), interleukin-6 (IL-6), tumor necrosis factors (TNF), and interferons (IFNs), which act as inflammatory inducers in a febrile response [4–6].

IL-1, a proinflammatory cytokine, is a one of the endogenous pyrogens (EPs) implicated in fever induction. EPs are able to circulate in the blood vessels and activate prostaglandin E2 (PGE_2_) synthesis in the brain via cAMP and other neurotransmitter activation, resulting in an elevation of the thermostatic set point and, in turn, an increase in heat loss and temperature [4, 7]. Moreover, IL-1 is also involved in the synthesis of prostaglandins (PGs) and lipid eicosanoids through cyclooxygenase enzyme (COX) activity. COX is a key enzyme that present two isoforms (COX-1 and COX-2), both of which act as rate-limiting enzymes in PG biosynthesis by metabolizing arachidonic acid. COX-1 is commonly expressed in various cells and tissues and facilitates housekeeping functions, whereas COX-2 is induced by proinflammatory cytokines, growth factors, infections, and other harmful stimuli. Additionally, COX-2 is one of the inflammatory markers, and it is associated with fever-related diseases [8–10]. In clinical practice, nonsteroidal anti-inflammatory drugs (NSAIDs), such as aspirin, indomethacin, diclofenac, and ibuprofen, are usually used to block COX activities and attenuate inflammatory responses, including fever [11, 12]. However, some toxicological effects stemming from the prolonged use of NSAIDs can cause side effects, such as hepatotoxicity, gastrointestinal irritation, renal impairment, and allergic reactions [13, 14].

Thai herbal Ha-Rak formula (HRF), also known as Bencha-Loga-Wichienis, is a polyherbal formula consisting of the roots of five medical plants:* Capparis micracantha* DC. (CM),* Clerodendrum petasites* S. Moore (CP),* Harrisonia perforat*e Merr. (HP),* Ficus racemosa* L. (FR), and* Tiliacora triandra* Diels (TT). It has been traditionally used as an antipyretic and anti-inflammatory drug for fever treatment, and it is included in the National List of Essential Medicines of Thailand. Previous* in vitro *and* in vivo* studies have indicated that HRF shows diverse pharmacological effects, including antioxidant, [15] anti-inflammatory [16], antipyretic and antinociceptive properties [17]. Additionally, certain components of HRF, namely, the extracts from FR and CP, have been shown to exert anti-inflammatory and antipyretic effects [18–20]. Nevertheless, the modulated effects of HRF on COX activity and prostaglandin synthesis are still imprecise and need clarification. We therefore investigated the regulation of HRF and its components on COX inhibition, using IL-1*β* induced in the human umbilical vein endothelial cell (HUVEC) model.

## 2. Materials and Methods

### 2.1. Reagents

All powders of HRF and its components were prepared by the Manufacturing Unit of Herbal Medicines and Products Ayurved Siriraj, Center of Applied Thai Traditional Medicine, Faculty of Medicine, Siriraj Hospital, Mahidol University. The human endothelial-SFM basal growth medium with L-glutamine was obtained from Gibco (Gibco, USA). The human COX-1 and COX-2 monoclonal antibodies and standards were purchased from Cayman Chemical (Ann Arbor, MI, USA). The ethanol reagent was purchased from Scharlau (Scharlau, Spain). The fetal bovine serum (FBS), penicillin, streptomycin, indomethacin, and other chemical reagents were purchased from Sigma-Aldrich (MO, USA).

### 2.2. Preparation of HRF and Its Component Extracts

The herbal powders were extracted with an 80% ethanol solution at a ratio of 1 : 10 (w/v). All of the HRF and its component extractions were evaporated under 40°C at a pressure within 110–180 mbar (Buchi, Switzerland) and kept in a minus 80°C freezer prior to lyophilization. The freeze-dried extracts were stored in the dark in a controlled temperature and humidity environment.

### 2.3. Human Umbilical Vein Endothelial Cell Isolation and Treatment

HUVECs were derived from umbilical cords obtained from normal, pregnant women, as previously described [21]. The isolated HUVECs were cultured in T-75 flasks with human endothelial-SFM basal growth medium and with L-glutamine (Gibco, USA) supplemented with fetal bovine serum 10% (FBS), penicillin (100 U/mL), and streptomycin (100 mg/mL), at 37°C in 5% CO_2_ in an incubator. When the cells were over 80% confluent, the HUVECs were treated with indomethacin (100 *µ*g/mL), HRF, and its component extracts at various concentrations prior to treatment with IL-1*β* (1 *η*g/mL); they were subsequently incubated at 37°C in 5% CO_2_ for 24 h.

### 2.4. Cell Viability Assay

MTT assay, as previously described [22], was conducted to determine the cytotoxicity of the test compounds. Briefly, HUVECs (3 × 10^4^ cells/well) were seeded on a 96-well plate and pretreated with HRF and the component extracts (0.00001–100 *µ*g/mL) for 24 h; after that, 200 *µ*L of MTT (200 *µ*g/mL) was added to each well and incubated for 1 h. To dissolve formazan, 100 *µ*L of DMSO solution was added to each well and measured using a spectrophotometer (SpectraMAX M5, Molecular Devices, CA) at an absorbance of 595 nm.

### 2.5. Real-Time Quantitative PCR Analysis (qRT-PCR)

The total RNA from each treatment was extracted with an Illustra RNA spin Mini RNA isolation kit (GE Healthcare, UK). All of the primer sequences are described in Table 1. The conditions are 95°C for 10 min and 95°C for 15 min, followed by 40 cycles of amplification at 60°C for 40 min, and, subsequently, at 72°C for 40 min. Analysis of the data was performed with the cycle threshold (Ct) method (ΔΔCt), normalized with the GAPDH gene used as a housekeeping gene and internal control.

### 2.6. Western Blot Analysis

HUVECs were treated with indomethacin, HRF, and the extracts (1, 10, and 100 *µ*g/mL) prior to IL-1*β* (1 *η*g/mL) stimulation for 24 h. The COX determination, including a Bradford protein assay, was performed as previously described [21]. Briefly, all samples were loaded into SDS-PAGE, underwent electrophoresis, and were transferred to nitrocellulose blotting membranes (Bio-Rad, Germany). After blocking with a solution of 5% skim milk for 1.5 h at room temperature, the membranes were incubated overnight with a specific monoclonal COX-1 or COX-2 antibody at 4°C and an anti-mouse IgG of COX-1 or anti-COX-2 (Sigma-Aldrich, USA, dilution 1/10000) for 1.5 h, respectively. *β*-Actin (Sigma-Aldrich, USA, dilution 1/5000) was used as an internal control in the experiment. The COX protein bands were visualized using VersaDoc™ Imaging Systems (Bio-Rad, Germany).

### 2.7. Determination of COX Activity

After treating the cells with the test compounds, the supernatant of each sample was collected after 24 h to measure the endogenous level of PGE_2_. To determine the exogenous PGE_2_ production, the medium from the HUVEC culture was discarded. The cells were washed with a phosphate-buffered saline (PBS) solution (138 mM NaCl; 2.7 mM KCl, 8 mM Na_2_HPO_4_; and 1.46 mM KH_2_PO_4_), incubated with a medium containing arachidonic acid (10 *µ*M) for 10 min. The level of PGE_2_ was measured by using an enzyme immunoassay kit (GE Healthcare, UK).

### 2.8. Statistical Analysis

Data were presented as mean ± standard error of the mean (SEM). All experiments were performed in triplicate, and their results were analyzed by one-way analysis of variance (ANOVA), followed by Dunnett's post hoc test using GraphPad Prism version 5 for Windows (GraphPad Software Inc., San Diego, CA, USA). The statistically significantly value was set at *p* < 0.05.

## 3. Results

### 3.1. Cell Viability Assessments

The HUVECs were treated with HRF and its components at the increasing concentrations of 0.0001, 0.001, 0.01, 0.1, 1, 10, and 100 *µ*g/mL (data not shown). At 100 *µ*g/mL, the cell viabilities were higher than 90%, except for the cells treated with* Ficus racemosa* L. (Table 2). This result suggests that no obvious cytotoxicity was observed in the HUVECs incubated with HRF at up to 100 *µ*g/mL.

### 3.2. Inhibitory Effects of HRF and Its Components on COX mRNA Expression

**Figure 1:**
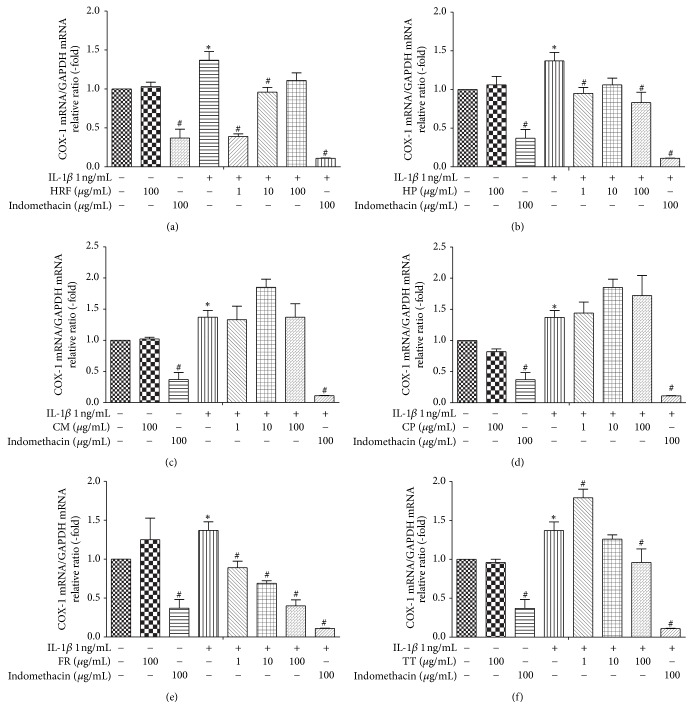
The effects of HRF (a) and its components: *Harrisonia perforata *Merr. (HP),* Capparis micracantha* DC. (CM),* Clerodendrum petasites* S. Moore (CP),* Ficus racemosa* L. (FR), and* Tiliacora triandra* Diels (TT) (b–f) (1, 10, and 100 *μ*g/mL) on COX-1 mRNA expression in HUVECs treated with IL-1*β* 1 ng/mL for 24 h. Control: nonaddition. ^*∗*^*p* < 0.05, versus control group; ^#^*p* < 0.05, versus IL-1*β* only.

**Figure 2:**
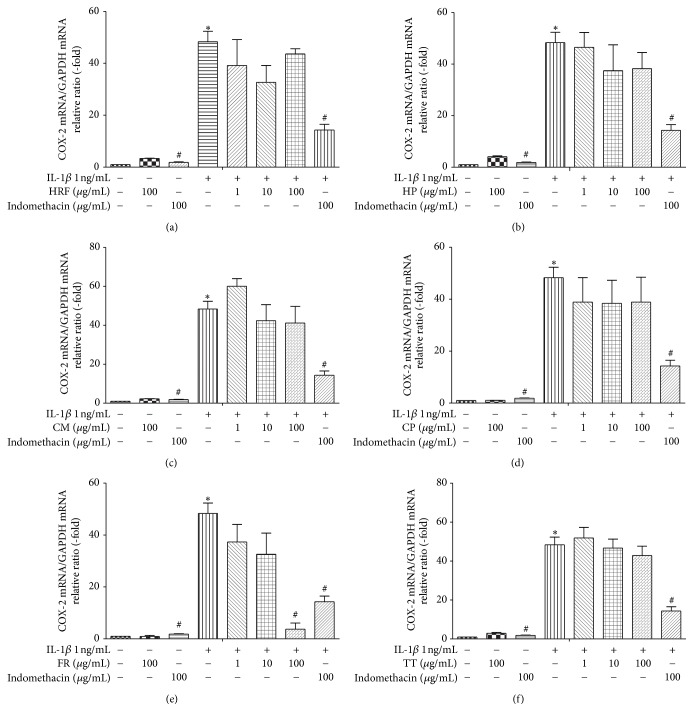
The effects of HRF (a) and its components:* Harrisonia perforata *Merr. (HP),* Capparis micracantha* DC. (CM),* Clerodendrum petasites* S. Moore (CP),* Ficus racemosa* L. (FR), and* Tiliacora triandra* Diels (TT) (b–f) (1, 10, and 100 *μ*g/mL) on COX-2 mRNA expression in HUVECs treated with IL-1*β* 1 ng/mL for 24 h measured by qRT-PCR. Control: nonaddition. ^*∗*^*p* < 0.05, versus control group; ^#^*p* < 0.05, versus IL-1*β* only.

The results demonstrated that IL-1*β* (1 *η*g/mL) significantly increased the COX1 and COX-2 mRNA expressions, relative to the untreated group of HUVECs (*p* < 0.05). Treatment with or without indomethacin (100 *μ*g/mL) in the IL-1*β*-induced HUVECs significantly attenuated the mRNA expressions of COX-1 and COX-2 (*p* < 0.05; Figures 1 and 2).

Furthermore, treatment with the HRF (1, 10 *μ*g/mL), TT (100 *μ*g/mL), and FR (1, 10, and 100 *μ*g/mL) extracts prior to the IL-1*β* challenge suppressed the COX-1 mRNA expression, with the FR extracts showing an inhibitory effect on COX-1 in a dose-dependent manner (Figures 1(a), 1(b), 1(e), and 1(f)). In addition, the highest dose of the FR extract (100 *μ*g/mL) provided the greatest inhibition of COX-2 mRNA expression induced by IL-1*β* stimulation (Figure 2(e)). However, the other treatment compounds (HRF, HP, CM, TT, and CP extracts) showed a tendency to decrease the COX-2 mRNA levels in HUVECs induced by IL-1*β* (Figures 2(a), 2(b), 2(c), and 2(d)).

### 3.3. HRF and Its Components Attenuated COX Protein Expression

**Figure 3:**
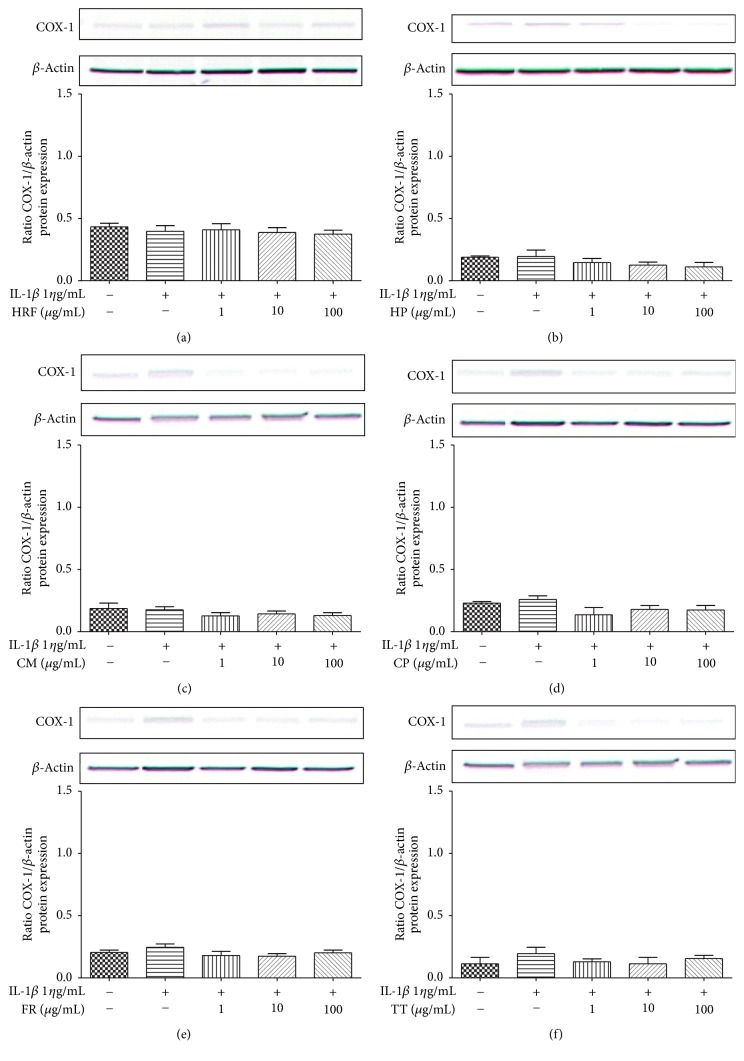
The effects of HRF (a) and its components:* Harrisonia perforata *Merr. (HP),* Capparis micracantha* DC. (CM),* Clerodendrum petasites* S. Moore (CP),* Ficus racemosa* L. (FR), and* Tiliacora triandra* Diels (TT) (b–f) on COX-1 protein expression in IL-1*β*-treated HUVECs for 24 h. COX protein was detected by Western blot. Control: nonaddition.

No statistically significant differences were observed in the COX-1 protein expression treated with HRF and its components prior to the IL-1*β* challenge (Figure 3).

**Figure 4:**
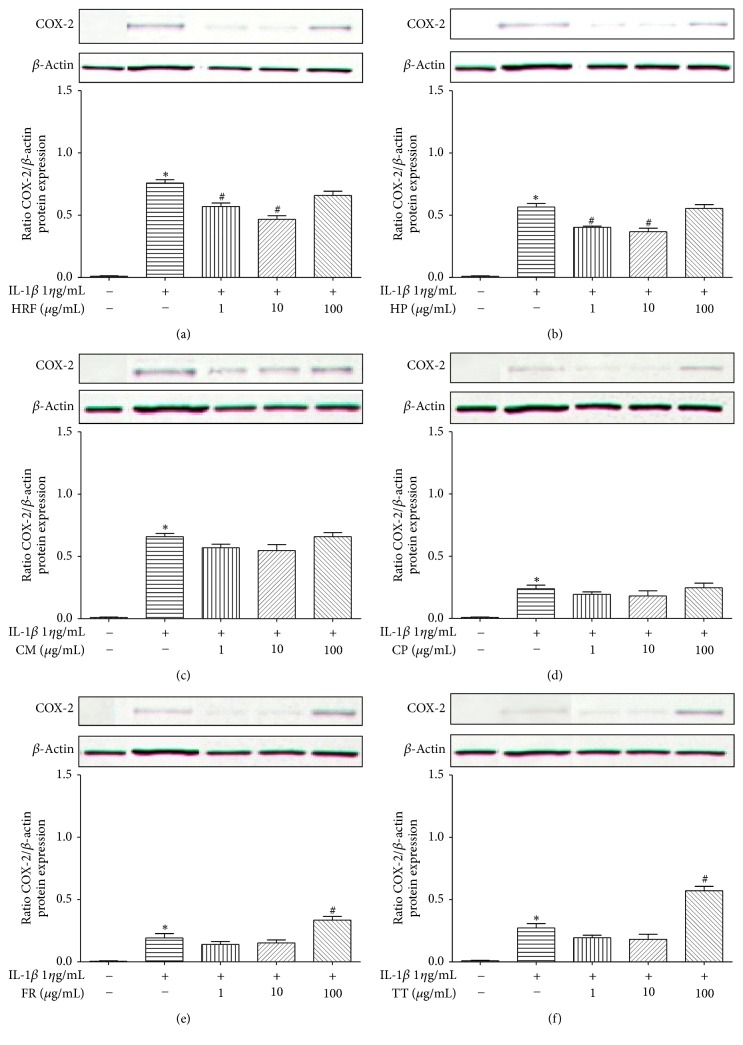
The effects of HRF (a) and its components:* Harrisonia perforata *Merr. (HP),* Capparis micracantha* DC. (CM),* Clerodendrum petasites* S. Moore (CP),* Ficus racemosa* L. (FR), and* Tiliacora triandra* Diels (TT) (b–f) on COX-2 protein expression in IL-1*β*-treated HUVECs for 24 h. COX protein was detected by Western blot. Control: nonaddition. ^*∗*^*p* < 0.05, versus control group; ^#^*p* < 0.05, versus IL-1*β* only.

IL-1*β* (1 *η*g/mL) noticeably induced COX-2 expression (*p* < 0.05) compared to the control groups (Figure 4). HRF (1 and 10 *μ*g/mL) and HP (1 and 10 *μ*g/mL) significantly decreased levels of COX-2 protein induced by IL-1*β*. However, the highest dose of HRF (100 *μ*g/mL) and HP (100 *μ*g/mL) extracts remarkably affected COX-2 protein inhibition (Figures 4(a) and 4(b)).

### 3.4. Effects of HRF and Its Components on COX Activity through Endogenous and Exogenous PGE_2_ Production

**Figure 5:**
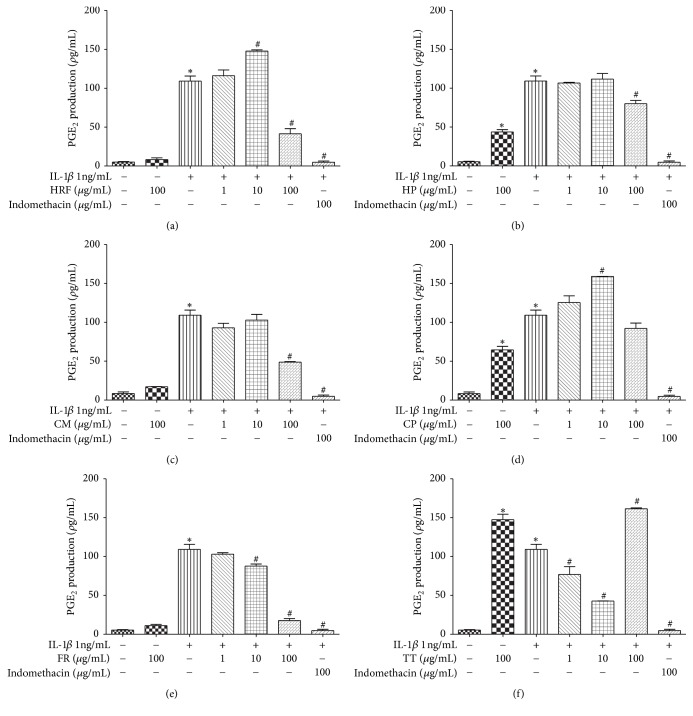
The effects of HRF (a) and its components:* Harrisonia perforata *Merr. (HP),* Capparis micracantha* DC. (CM),* Clerodendrum petasites* S. Moore (CP),* Ficus racemosa* L. (FR) and* Tiliacora triandra* Diels (TT) (b–f) on COX activity in the presence of endogenous AA in IL-1*β*-treated HUVECs. The data represent mean ± SEM of three experiments. Control: nonaddition. ^*∗*^*p* < 0.05, versus control group; ^#^*p* < 0.05, versus IL-1*β* only.

HRF (100 *µ*g/mL), HP (100 *µ*g/mL), CM (100 *µ*g/mL), FR (10 and 100 *µ*g/mL), and TT (1 and 10 *µ*g/mL) extracts significantly restrained endogenous PGE_2_ production (Figures 5(a)–5(c), 5(e), and 5(f)). Meanwhile, HRF (10 *µ*g/mL) and CP (10 *µ*g/mL) noticeably increased PGE_2_ accumulation (Figures 5(a) and 5(d)) in IL-1*β* induced HUVECs (*p* < 0.05).

**Figure 6:**
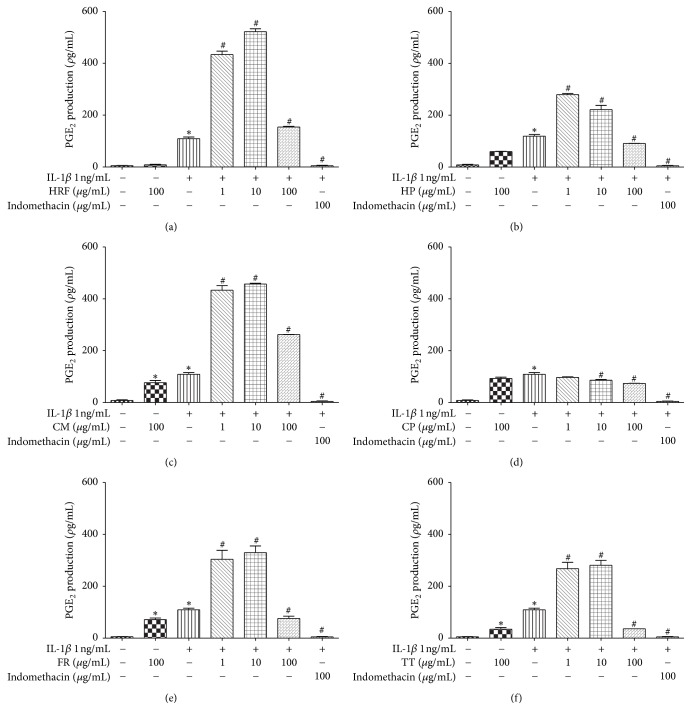
The effects of HRF (a) and its components:* Harrisonia perforata *Merr. (HP),* Capparis micracantha* DC. (CM),* Clerodendrum petasites* S. Moore (CP),* Ficus racemosa* L. (FR), and* Tiliacora triandra* Diels (TT) (b–f) on COX activity in the presence of exogenous AA in IL-1*β*-treated HUVECs. The data represent mean ± SEM of three experiments. Control: nonaddition. ^*∗*^*p* < 0.05, versus control group; ^#^*p* < 0.05, versus IL-1*β* only.

As for exogenous PGE_2_ generation, the results indicated that all concentrations of the test compounds, including HRF and its components, significantly enhanced COX activity, whereas CP (10 and 100 *µ*g/mL) significantly restrained exogenous PGE_2_ production (Figure 6).

## 4. Discussion

We have presented the modulatory effects of HRF and its components by focusing on the COX-PGE_2_ pathway related to the febrile response in the HUVEC model. Several scientific reports have indicated that many cytokines and other mediators, including IL-1*β*, play a critical role in fever induction through increased levels of PGE_2_ in the hypothalamic thermoregulatory center [23]. Moreover, the COX-PGE_2_ pathway is also responsible for inflammatory response development [24]. Cyclooxygenase enzymes (COXs), comprising two isoforms (COX-1 and COX-2), are the key enzymes in prostaglandin generation [25]. The induction of COX-2 by several proinflammatory cytokines represents an important mechanism controlling the overall production of prostanoids and the evolution of the inflammatory response [26]. Our results also confirm a previous report that IL-1*β* can activate COX-2 mRNA and protein expressions and can consequently generate PGE_2_ production in the HUVEC model [27]. In this study, we also observed the effect of IL-1*β* on endogenous and exogenous PGE_2_ releases through COX-2 expression in HUVECs. We found that IL-1*β* could mediate endogenous and exogenous PGE_2_ production through COX-2 metabolites [26]. Thus, the anti-inflammatory effect is probably due to IL-1*β*'s ability to inhibit the COX-2 enzyme [28]. After the endogenous PGE_2_ production was measured, exogenous AA was used to treat the cells. The amount of PGE_2_ measured in this media reflects the level of PGE_2_ synthesis by the exogenous AA and also the COX activity [29]. Interestingly, HRF significantly enhanced exogenous AA, leading to the upregulation of COX-2 expression induced by IL-1*β* (Figure 6(a)). These results suggest that the use of exogenous AA as substrates will help to evaluate the COX enzyme directly [30].

In this study, HRF and its components did inhibit the COX-2 protein expression in HUVECs (Figure 4), but the amount of COX-1 protein expression was not affected. The predicted modulatory effects of HRF present in the protein clearly suggest its potential of being an inhibitor of COX-2. Our findings demonstrate that the formula and some components can modulate COX isoforms, as found in previous reports; for instance,* Clerodendrum petasites* S. Moore showed an inhibition of COX-2-mediated. PGE_2_ production* in vitro *[31] and a bioassay-guided fractionation of the ethanol extract of* Ficus racemosa* L. showed potent inhibitory activity against both COX-1 and 5-LOX* in vitro* [19].

**Figure 7:**
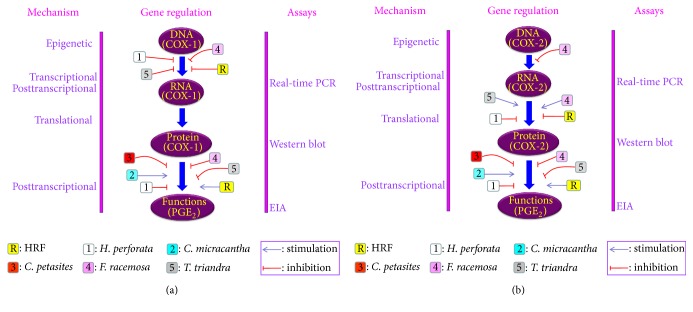
Summary of the effects of HRF and its components on COX-1 (a) and COX-2 (b) gene regulation pathway in HUVECs.

Our findings provide preliminary evidence that HRF and its components can modulate the COX enzymes directly involved with PGE_2_ production in IL-1*β*-induced in HUVECs (Figure 7).

However, further studies to elucidate HRF's pharmacological effects and other signaling pathways involving its anti-inflammatory and antipyretic potentials should be investigated to support its traditional usage and clinical application and to develop HRF as a novel antipyretic agent in the future.

## Figures and Tables

**Table 1:** List of primer sequences.

Primer name	GenBank	Sense primer (5′ → 3′)	Anti-sense primer (5′ → 3′)
COX-1	NM_001271368.1	GACCCGCCTCATCCTCATAG-3	CCACCGA TCTTGAAGGAGTCA
COX-2	NM_006662.2	CAAAAGCTGGGAAGCCTTCT	CCATCCTTGAAAAGGCGCAG
GAPDH	NM_001289746.1	GACCACTTTGTCAAGCTCATTTCC	TGAGGGTCTCTCTCTTCCTCTTGT

COX-1: cyclooxygenase-1; COX-2: cyclooxygenase-2; GAPDH: glyceraldehyde-3-phosphate dehydrogenase.

**Table 2:** Cell viability of 100 *µ*g/mL of HRF and its components against HUVECs.

Herbal	Cell viability (%)
Thai herbal Ha-Rak formula	94 ± 5
*Harrisonia perforat*e Merr.	120 ± 4
*Capparis micracantha* DC.	125 ± 3
*Clerodendrum petasites* S. Moore	99 ± 2
*Ficus racemosa* L.	58 ± 4
*Tiliacora triandra* Diels	99 ± 4

The data represent mean ± SEM of triplicate wells from at least 3 separate experiments performed on different days.
